# Circulating secretory IgA antibodies against cyclic citrullinated peptides in early rheumatoid arthritis associate with inflammatory activity and smoking

**DOI:** 10.1186/s13075-016-1014-1

**Published:** 2016-05-23

**Authors:** Karin Roos, Klara Martinsson, Michael Ziegelasch, Yngve Sommarin, Anna Svärd, Thomas Skogh, Alf Kastbom

**Affiliations:** Division of Rheumatology, Department of Clinical and Experimental Medicine, Linköping University, Linköping, Sweden; Euro Diagnostica AB, Malmö, Sweden; Rheumatology Clinic, Falun Hospital, Falun, Sweden

**Keywords:** Rheumatoid arthritis, Anticitrullinated protein antibodies, Secretory immunoglobulin A, Mucosal immunity

## Abstract

**Background:**

A possible association between mucosal immunization and inflammation, as well as the initiation and propagation of rheumatoid arthritis (RA), is attracting renewed interest. The aim of this study was to evaluate the possible occurrence and clinical correlations of circulating secretory immunoglobulin A (SIgA) antibodies against the second-generation cyclic citrullinated peptides (CCP) among patients with recent-onset RA followed prospectively over 3 years.

**Methods:**

Baseline serum samples from 636 patients with recent-onset RA were analyzed for SIgA anti-CCP antibodies by using an enzyme-linked immunosorbent assay with a secondary antibody directed against secretory component. SIgA anti-CCP status at baseline was analyzed in relation to smoking, HLA-DRB1/shared epitope (SE), and the disease course over 3 years. Significant findings were evaluated in regression analysis that included age, sex, smoking, and SE.

**Results:**

Seventeen percent of the patients tested positive for circulating SIgA anti-CCP, and the occurrence was confirmed by detection of secretory component in an affinity-purified IgA anti-CCP fraction. SIgA anti-CCP positivity at baseline was associated with slightly higher baseline erythrocyte sedimentation rate (ESR) (mean 38 vs. 31 mm/first hour, *p* = 0.004) and C-reactive protein (CRP) (mean 30 vs. 23 mg/L, *p* = 0.047). During follow-up, SIgA anti-CCP-positive patients had a higher mean AUC regarding ESR (adjusted *p* = 0.003), although there were no significant differences regarding CRP, tender and swollen joint counts, or radiological joint damage (median Larsen progression 1.0 vs. 1.0, *p* = 0.22). SIgA anti-CCP was associated significantly with smoking (79 % ever smokers among SIgA anti-CCP-positive patients vs. 59 % in SIgA anti-CCP-negative patients, adjusted OR 2.19, 95 % CI 1.01–4.37, *p* = 0.027) but not with carriage of the SE (80 % vs. 73 %, *p* = 0.62).

**Conclusions:**

Circulating SIgA anti-CCP, which is present in a subgroup of patients with early RA, is not related to SE, but it is environmentally linked to cigarette smoking. This finding strengthens the hypothesis that immunization against citrullinated peptides and/or proteins may occur at mucosal surfaces of the airways. Analysis of SIgA antibodies in serum may be a convenient and more versatile means to investigate the “mucosal connection” in RA compared with analyses in mucosal fluid samples.

## Background

Anti-citrullinated protein antibodies (ACPA) of both the IgG and IgA classes are commonly found in both synovial fluid and the circulation of patients with rheumatoid arthritis (RA) [1, 2], and they are highly specific for the disease [3]. The occurrence of circulating immunoglobulin A (IgA), IgM, and/or IgG ACPA often precedes a clinical diagnosis of RA [4, 5], but it is seldom induced after disease onset [6, 7]. Although a large number of citrullinated autoantigens have been described, the most commonly used ACPA test detects IgG class antibodies to synthetic cyclic citrullinated peptides (CCP).

In recent years, the possible association between mucosal immunity and the initiation and propagation of RA has attracted renewed interest. For instance, chronic inflammation of the periodontium (i.e., periodontitis [PD]) associates with ACPA-positive RA, and patients with both conditions have more severe disease than patients with RA without PD [8, 9]. Also, IgG class serum antibodies to the PD-associated bacterium *Porphyromonas gingivalis* have been shown to correlate with serum levels of IgM and IgG2 anti-CCP levels [10] as well as with more active early disease [11]. IgG anti-CCP may be found in fluid from gingival crevices of patients with PD [12], and IgA anti-CCP antibodies have been demonstrated in saliva from patients with RA [13].

Involvement of airway mucosal surfaces in ACPA induction, and subsequent RA development is frequently brought forward, originally based on the epidemiological connection between inhaled toxic agents (e.g., cigarette smoke and silica) and an increased risk of ACPA-positive RA [14–16]. Further support is provided by the findings of ACPA enrichment in sputum and bronchoalveolar fluid [17, 18] as signs of local autoantibody production, as well as by the identification of identical citrullinated autoantigens in both lungs and joints of patients with RA [19]. Also, radiological parenchymal abnormalities of the lungs are more common in ACPA-positive individuals compared with those who are ACPA-negative, regardless of smoking and RA status [17, 20].

Mucosal immunity of the gastrointestinal tract regained attention when it was shown in the early 1990s that induction of oral tolerance to type II collagen could alleviate arthritis in mice and humans, although later the therapeutic effect in humans was found to be disappointing [21]. Later work has been focused on interactions with the gut microbiome, where manipulations of the intestinal microbiota were shown to influence arthritis severity in several animal models [22, 23]. Interestingly, patients with RA have been reported to have an altered fecal microbiota compared with disease controls [24], and anti-CCP antibodies and increased total secretory IgA (SIgA) levels have been demonstrated in feces [25].

SIgA is produced at mucosal surfaces, but it can also be detected in low concentrations in the systemic circulation [26]. In contrast to circulating IgA, which is mostly monomeric, SIgA is mainly dimeric and complexed with a secretory component (SC) (i.e., a remnant of the polymeric immunoglobulin receptor responsible for the active transport of antibodies across mucosal membranes) [27]. Eijgenraam et al. reported antigen-specific SIgA in serum after mucosal immunization with cholera toxin subunit B [28]. Thus, mucosal immunization in autoimmune diseases could potentially be investigated by analysis of SIgA autoantibodies in serum, enabling more convenient sample handling, more reliable quantitative analyses, and access to larger patient cohorts compared with what is achievable regarding mucosal secretions.

Before the discovery of ACPA, rheumatoid factor (RF) was the predominant serologic marker of RA. RF of IgA class has repeatedly been associated with smoking and with more severe disease [29–31]. Also, RF complexed with SC has been detected in RA, but the predictive value of these antibodies was not evaluated [32]. The aim of this study was to detect SIgA anti-CCP in sera from patients with RA and to determine its relationship to disease course, cigarette smoking, and genetic (HLA-DRB1) risk factors.

## Methods

### Study subjects

Two prospective Swedish early RA cohorts, designated “timely interventions in RA” (TIRA), formed the basis of the present study [33]. The prerequisites for inclusion were symptom duration ≥6 weeks but <12 months since the first joint swelling as judged by the patient, as well as the following:

- Fulfillment of at least four of seven of the 1987 revised American College of Rheumatology criteria for RA [34] (*n* = 605 [95.1 %]) *or*
- Morning stiffness for ≥60 minutes *and* symmetrical arthritis *and* small joint arthritis (fingers, wrists, or toes) (*n* = 31 [4.9 %])

TIRA-1 patients were enrolled between 1996 and 1998 and prospectively followed with longitudinal assessments of 28-joint Disease Activity Score (DAS28) [35], erythrocyte sedimentation rate (ESR), C-reactive protein (CRP), the Swedish version of the Health Assessment Questionnaire [36], and information regarding ongoing disease-modifying antirheumatic drugs(DMARDs). Smoking habits were assessed by questionnaires as previously described [37].

TIRA-2 patients were enrolled between 2006 and 2008 with identical inclusion criteria and follow-up procedures as those used for TIRA-1, except that yearly radiographs of the hands and feet from baseline were obtained and graded for joint damage according to the Larsen score [38]. Smoking habits were assessed either with a questionnaire provided by the Epidemiological Investigations of RA Study (*n* = 207) [39] or by chart review (*n* = 58).

Table 1 summarizes the baseline characteristics of both cohorts. Baseline serum samples were available from 197 (62.1 %) of 317 TIRA-1 patients and from 439 (97.1 %) of 452 TIRA-2 patients. There were no significant differences in clinical characteristics between patients who had samples available compared with those without. All patients gave their written informed consent to participate, and the ethics committee in Linköping, Sweden, approved the study protocols.

**Table 1:** Baseline characteristics

Characteristics	TIRA cohorts (*n* = 636)
Women	426/636 (67.0 %)
Mean age, years (SD)	57.6 (15.0)
Median baseline Larsen score^a^ (IQR)	2.0 (4.3)
RF-positive	387/636 (60.8 %)
IgG anti-CCP-positive	421/622 (67.7 %)
IgA anti-CCP-positive	251/635 (39.5 %)
SIgA anti-CCP-positive	110/636 (17.3 %)
Median baseline HAQ (IQR)	0.9 (0.6)
Any baseline DMARD	504/631 (79.9 %)

*TIRA* Swedish acronym for “timely interventions in rheumatoid arthritis”, *CCP* cyclic citrullinated peptides, *RF* rheumatoid factor, *DMARD* disease-modifying antirheumatic drug, *IQR* interquartile range, *IgG* immunoglobulin G, *IgA* immunoglobulin A, *SIgA* secretory immunoglobulin A, *HAQ* Health Assessment Questionnaire

Values are number (%) of patients unless otherwise indicated

^a^Data available from 398 patients

### Isotype-specific enzyme-linked immunoassays

Serum samples (*n* = 636) taken at inclusion were analyzed for anti-CCP of SIgA isotype using a modification of the commercially available IgG class anti-CCP2 enzyme-linked immunosorbent assay (ELISA) (CCPlus® Immunoscan; Euro Diagnostica AB, Malmö, Sweden). Serum samples were stored at −70 °C until analysis. The sera were diluted 1:25 with kit buffer and added in duplicate wells. An RA reference serum with high SIgA anti-CCP level was applied in dilution series (1:12.5 to 1:800) to achieve a standard curve and subsequent conversion of the results into arbitrary units (AU) per milliliter. Incubations and rinsing were done according to the kit manufacturer’s guidelines. A polyclonal goat IgG antihuman secretory component (GAHU/SC/PO; Nordic BioSite, Täby, Sweden) diluted 1:2000 in kit buffer was used as a secondary antibody. Absorbance was read by spectrophotometry at 450 nm (TECAN Sunrise software, Magellan V7.1; Tecan Nordic AB, Mölndal, Sweden). IgG and IgA anti-CCP antibodies in TIRA-1 were analyzed by ELISAs as previously described [6, 40] and in TIRA-2 by ELISA for IgG anti-CCP (CCPlus® Immunoscan) and by fluorescence enzyme immunoassays on the Phadia 250 instrument (EliA; Thermo Fisher AB, Uppsala, Sweden) for IgA anti-CCP. The cutoffs for a positive SIgA anti-CCP antibody test were set to 153 AU/ml and to ≥2 μg/L for the IgA anti-CCP EliA, both above the 99th percentile among 101 healthy blood donors. Regarding IgG anti-CCP antibodies, we used the cutoff level suggested by the manufacturer (25 AU/ml). Citrulline specificity of the SIgA anti-CCP method was tested by analyzing ten RA serum samples on microtiter plates coated with cyclic arginine peptides (CAP) (Euro Diagnostica AB) as well as on plates coated with CCP. In total, 621 of 636 serum samples were analyzed regarding all 3 anti-CCP isotypes (IgG, IgA, and SIgA).

### Antibody affinity purification

Anti-CCP antibodies of IgA and IgG class were isolated from a highly positive serum sample by affinity chromatography using aCCP column (Euro Diagnostica AB). The sample was filtered through a 0.2-μm pore size filter and then added to the column. Bound antibodies were eluted using 0.1 M glycine (pH 2.7) and immediately neutralized with 1 M Tris (pH 9.0). The CCP-specific antibodies were then added to a protein G column (Pierce Biotechnology, Rockford, IL, USA), and the IgG class anti-CCP antibodies were eluted in 0.3-ml aliquots using IgG elution buffer (Pierce Biotechnology). The flow-through (containing IgA class anti-CCP antibodies) was added to a Peptide M column (InvivoGen, San Diego, CA, USA), and the IgA class anti-CCP antibodies were eluted (in 0.3-ml aliquots) using 0.1 M sodium acetate (pH 4.0). Immediately after elution, both IgG and IgA class anti-CCP antibodies were neutralized with 1 M Tris-HCl (pH 8.3). The purified antibodies were stored at −20 °C until further use.

### Western blot analysis

The samples were mixed 50:50 with Laemmli buffer containing 2-β-mercaptoethanol and heated to 95 °C for 5 minutes. The purified anti-CCP antibodies were diluted to 0.14 μg/ml, and 35 μl of each sample was added to the wells of a 10 % SDS-PAGE gel (Bio-Rad Laboratories, Hercules, CA, USA), which was run for 90 minutes at 180 V (PowerPac HC; Bio-Rad Laboratories). Precision Plus Protein WesternC Standard (Bio-Rad Laboratories) was used as a molecular weight marker. Following electrophoresis, the antibodies were transferred to a nitrocellulose membrane (Bio-Rad Laboratories) presoaked in Towbin buffer. Blotting was carried out using the Bio-Rad PowerPac HC for 30 minutes at 80 V. The membrane was washed and blocked using 5 % fat-free milk powder (Bio-Rad Laboratories) for 1 h. After blocking, the membrane was placed in Tris-buffered saline (TBS) with 0.05 % Tween-20 (TBS-T) for 5 minutes. The molecular weight marker was cut out and transferred to a container with the detection antibody (Bio-Rad Laboratories) diluted 1:60,000 in TBS-T and incubated for 30 minutes, followed by washing in TBS-T and TBS. The rest of the membrane was transferred to a container with the SIgA detection antibody (GAHU/SC/PO; Nordic BioSite) diluted to 1:50,000 in TBS-T and incubated overnight at 4 °C. Thereafter, the membrane was washed in TBS-T and TBS, mounted and incubated for 1 minute with the substrate (ECL Prime Western Blotting Detection Reagent; GE Healthcare Life Sciences, Little Chalfont, UK). The membrane was exposed for 1 minute (high-performance chemiluminescence film; GE Healthcare Life Sciences), and the film was developed and fixed using D-19 Silver (Kodak, Rochester, NY, USA) and Fixer (Kodak).

### Genetic analyses

In TIRA-1, genotyping of HLA-DRB1 was performed by polymerase chain reaction amplification with sequence-specific primers (GenoVision, Oslo, Norway); in TIRA-2, it was carried out by Sanger sequencing (BGI Clinical Laboratories, Shenzhen, China). In both cohorts, shared epitope (SE) was defined as HLA-DRB1*01, *0401, *0404, *0405, *0408, *0409, *0410, *0413, *0416, *0419, *0421, or *10.

### Statistics

Clinical and laboratory measurements of disease severity (e.g., ESR, CRP, and DAS28) were compared by using Student’s *t* test at baseline, and during follow-up by calculating AUC for months 0–36. Missing values were assumed to occur at random, and, unless this occurred during the more dynamic first 6 months (which resulted in exclusion from the analysis), we adopted the last observation carried forward, which occurred in 4.4 % of the occasions. Student’s *t* test was used to compare AUC between patients testing positive versus negative regarding anti-CCP antibodies of each isotype. Linear regression analysis was performed to evaluate the association between SIgA anti-CCP and ESR adjusted for age, sex, and carriage of SE. As IgG anti-CCP is well known to associate with the disease course in early RA, and since SIgA/IgA anti-CCP antibodies almost exclusively occur in IgG anti-CCP-positive patients, we chose to evaluate the influence of SIgA and IgA anti-CCP antibodies on disease course and pharmacotherapy in IgG anti-CCP-positive patients only.

The Mann-Whitney *U* test was applied to evaluate differences between anti-CCP levels and radiological joint damage. Spearman´s correlation (ρ) was used to evaluate correlations between levels of SIgA anti-CCP, other ACPA isotypes, and baseline disease activity measures. Fisher’s exact test was performed to test differences in the occurrence of SE and smoking. Also, the association between smoking and SIgA anti-CCP was tested in a logistic regression analysis, adjusting for age, sex, and carriage of SE. Statistical analyses were performed using IBM SPSS 22.0 software (IBM, Armonk, NY, USA), and two-sided *p* values less than 0.05 were considered significant.

## Results

### Occurrence of SIgA anti-CCP

**Fig. 1:**
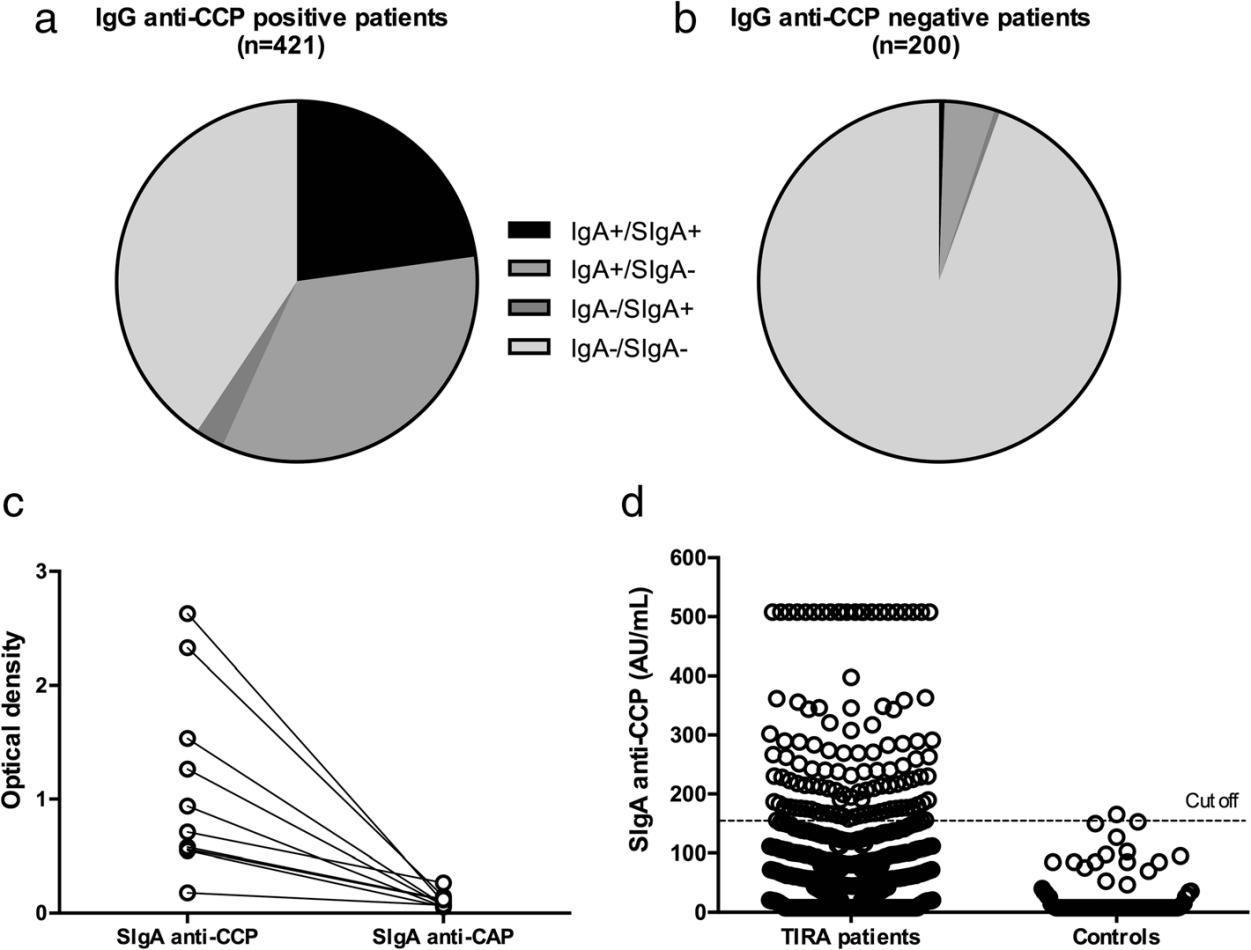
Occurrence of immunoglobulin A (IgA) and secretory IgA (SIgA) anti-cyclic citrullinated protein (anti-CCP) antibodies in patients with early rheumatoid arthritis (RA) testing positive (**a**) or negative (**b**) for IgG anti-CCP. SIgA reactivity to CCP and cyclic arginine peptide (CAP) in ten patients with RA (**c**), and SIgA anti-CCP levels in patients with RA and control subjects (**d**). *AU* arbitrary units

As measured by ELISA, circulating SIgA anti-CCP occurred in 29 (14.7 %) TIRA-1 patients and 81 (18.5 %) TIRA-2 patients, respectively (17.3 % occurrence in total) (Fig. 1a–d). Among patients with IgG anti-CCP antibodies, 25.4 % also had SIgA anti-CCP antibodies, while IgA anti-CCP antibodies occurred in 38.6 % (Fig. 1a, b). SIgA anti-CCP and IgA anti-CCP were both rare in the absence of IgG anti-CCP antibodies (occurring in 2 and 10 patients, respectively). Ninety-six (15.5 %) of six hundred twenty-one of the patients tested positive for all analyzed isotypes.

Citrulline-dependent specificity of the SIgA antibodies was evaluated by testing the reactivity to CAP control plates in ten anti-CCP-positive serum samples. The median optical density (OD) for SIgA anti-CAP was 0.09 (range 0.05–0.27), and the median OD for SIgA anti-CCP was 0.83 (range 0.18–2.63) for the same serum samples (Fig. 1c). No SIgA anti-CAP value exceeded 40 % of the corresponding SIgA anti-CCP OD. SIgA anti-CCP levels correlated significantly with IgG anti-CCP (ρ = 0.70, *p* < 0.001) and with levels of IgA anti-CCP (ρ = 0.69 in TIRA-1 and ρ = 0.64 in TIRA-2, *p* < 0.001 for both; cohorts presented separately because of different detection methods used).

**Fig. 2:**
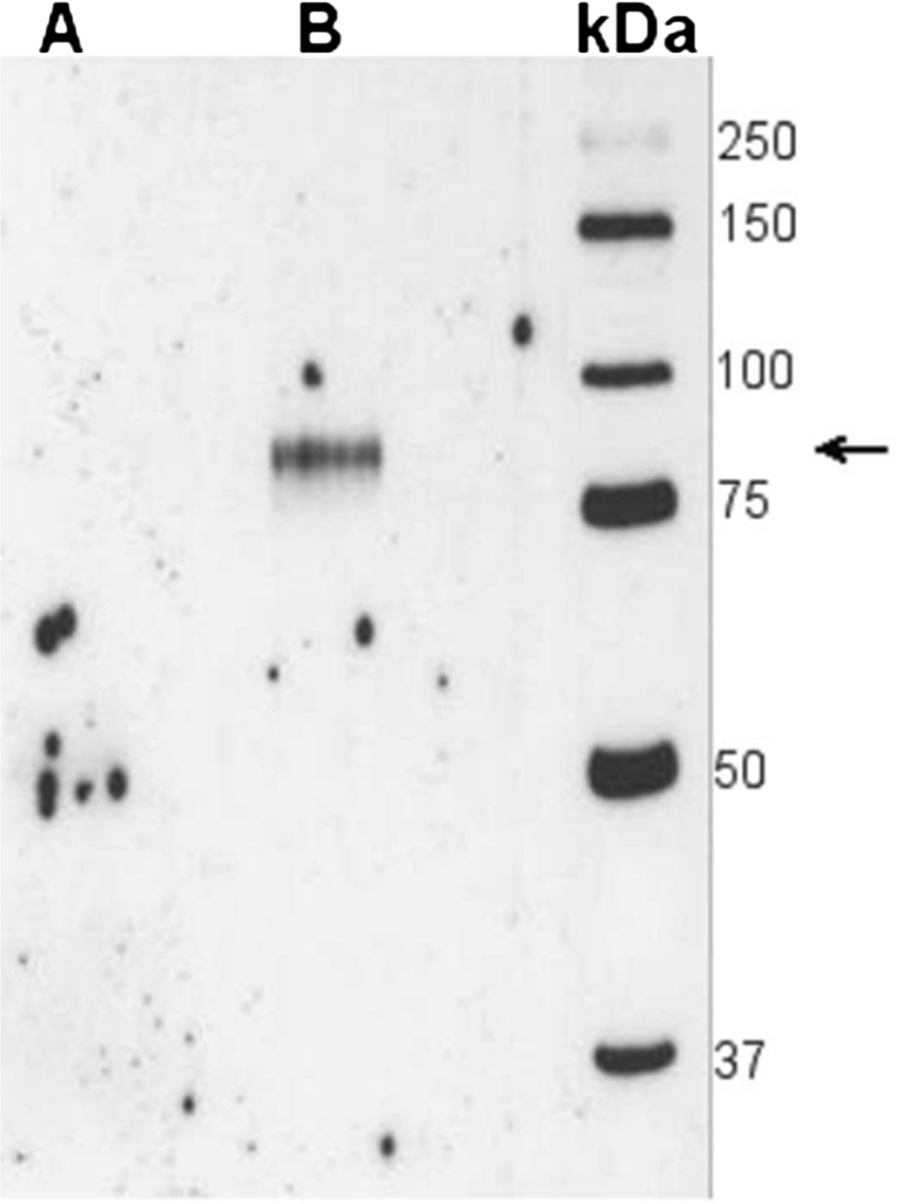
Western blotting for the detection of secretory component in an (*A*) immunoglobulin G (IgG) anti-cyclic citrullinated protein (anti-CCP) antibody fraction and (*B*) an IgA anti-CCP fraction. An 80 kDa band corresponding to the secretory component is visible in the IgA anti-CCP fraction

To confirm the presence of circulating SIgA anti-CCP, we performed Western blot analysis to detect SC in affinity-purified anti-CCP antibodies from IgG and IgA fractions of patient sera. In the IgA anti-CCP eluate, an 80 kDa band appeared, corresponding to the molecular weight of SC [27], while no band was visible in the IgG anti-CCP fraction (Fig. 2).

### Circulating SIgA anti-CCP and disease outcome

A modest but statistically significant correlation was observed between levels of SIgA anti-CCP and ESR at baseline (ρ = 0.09, *p* = 0.02), while the number of tender and swollen joints at baseline tended to be inversely correlated with SIgA anti-CCP levels (ρ = −0.12, *p* = 0.002, and ρ = −0.08, *p* = 0.038, respectively). There were no significant correlations regarding baseline DAS28 (ρ = −0.06, *p* = 0.15) or CRP (ρ = −0.02, *p* = 0.65).

As IgG anti-CCP antibody status is known to predict the disease course [6] and therefore may influence therapeutic decisions in early RA, and since SIgA anti-CCP almost exclusively occurs in IgG anti-CCP-positive patients, we chose to perform analyses of disease severity only among the IgG anti-CCP-positive patients. Patients testing positive regarding SIgA (and IgG) anti-CCP had higher mean baseline CRP and ESR levels than the patients negative for SIgA (but still positive regarding IgG) anti-CCP (CRP 29.9 vs. 22.7 mg/L, *p* = 0.047; ESR 38.0 vs. 30.8 mm/first hour, *p* = 0.004). There were no significant differences between the two groups regarding baseline DAS28 (5.1 vs. 5.1, *p* = 0.57), tender joint count (7.1 vs. 7.9, *p* = 0.24), or swollen joint count (8.1 vs. 7.9, *p* = 0.73). Patients testing positive for SIgA anti-CCP had higher baseline levels of both IgG and IgA anti-CCP than SIgA-negative patients (median IgG in TIRA-1 1270 vs. 424 AU/ml, *p* < 0.001; median IgA in TIRA-1 77.5 vs. 58.5 AU/ml, *p* < 0.001; median IgA in TIRA-2 10.5 vs. 5.5 μg/L, *p* < 0.001).

**Fig. 3:**
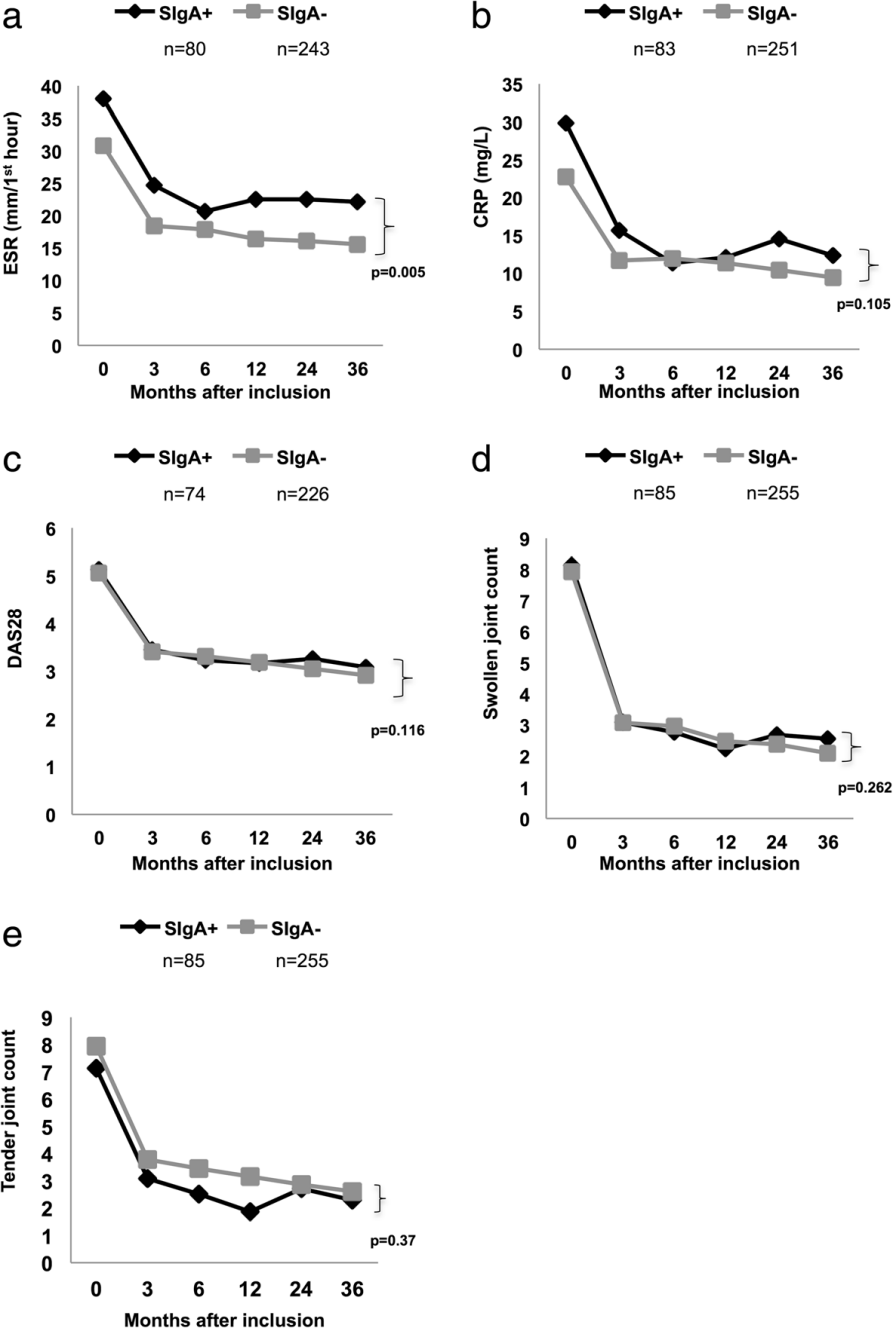
Three-year disease course of early rheumatoid arthritis in relation to secretory immunoglobulin A (SIgA) anti-cyclic citrullinated peptide (anti-CCP) antibody status as mirrored by (**a**) erythrocyte sedimentation rate (ESR), (**b**) C-reactive protein (CRP), (**c**) 28-joint Disease Activity Score (DAS28), (**d**) swollen joint count, and (**e**) tender joint count. Mean values are shown, and *p* values refer to differences in AUC

As seen in Fig. 3, comparison of AUC for the 3-year follow-up period revealed significantly higher mean ESR among SIgA-positive patients (*p* = 0.005) but no significant differences regarding CRP, DAS28, and swollen or tender joint count. In univariate analyses of IgG anti-CCP-positive patients, SIgA anti-CCP status and age (but not sex, smoking, or SE) were significantly associated with AUC ESR. In a linear regression analysis adjusting for age, SIgA anti-CCP positivity remained significantly associated with higher mean AUC for ESR (*p* = 0.003). In a similar model, but analyzing absolute values at the 3-year follow-up, SIgA anti-CCP positivity was associated with a 5.0 mm/first hour increase in ESR (95 % CI 0.7–9.2) compared with SIgA anti-CCP-negative patients.

IgA anti-CCP status was evaluated using the same approach, and no significant differences were detected in AUC of the above-mentioned disease activity measures (data not shown). The proportion of patients prescribed DMARD or nonsteroidal anti-inflammatory drug (NSAID) treatment did not differ between SIgA-positive and SIgA-negative patients at any of the follow-up visits (data not shown). Radiological joint damage in the TIRA-2 cohort did not significantly differ between SIgA anti-CCP-positive and SIgA anti-CCP-negative patients at baseline (median Larsen score 2.0 vs. 2.0, *p* = 0.61) or at the 3-year follow-up visit (2.5 vs. 4.0, *p* = 0.42). Likewise, there was no difference in median Larsen score progression over 3 years (1.0 vs. 1.0, *p* = 0.22).

### Circulating SIgA anti-CCP in relation to HLA-DRB1/shared epitope and smoking habit

**Fig. 4:**
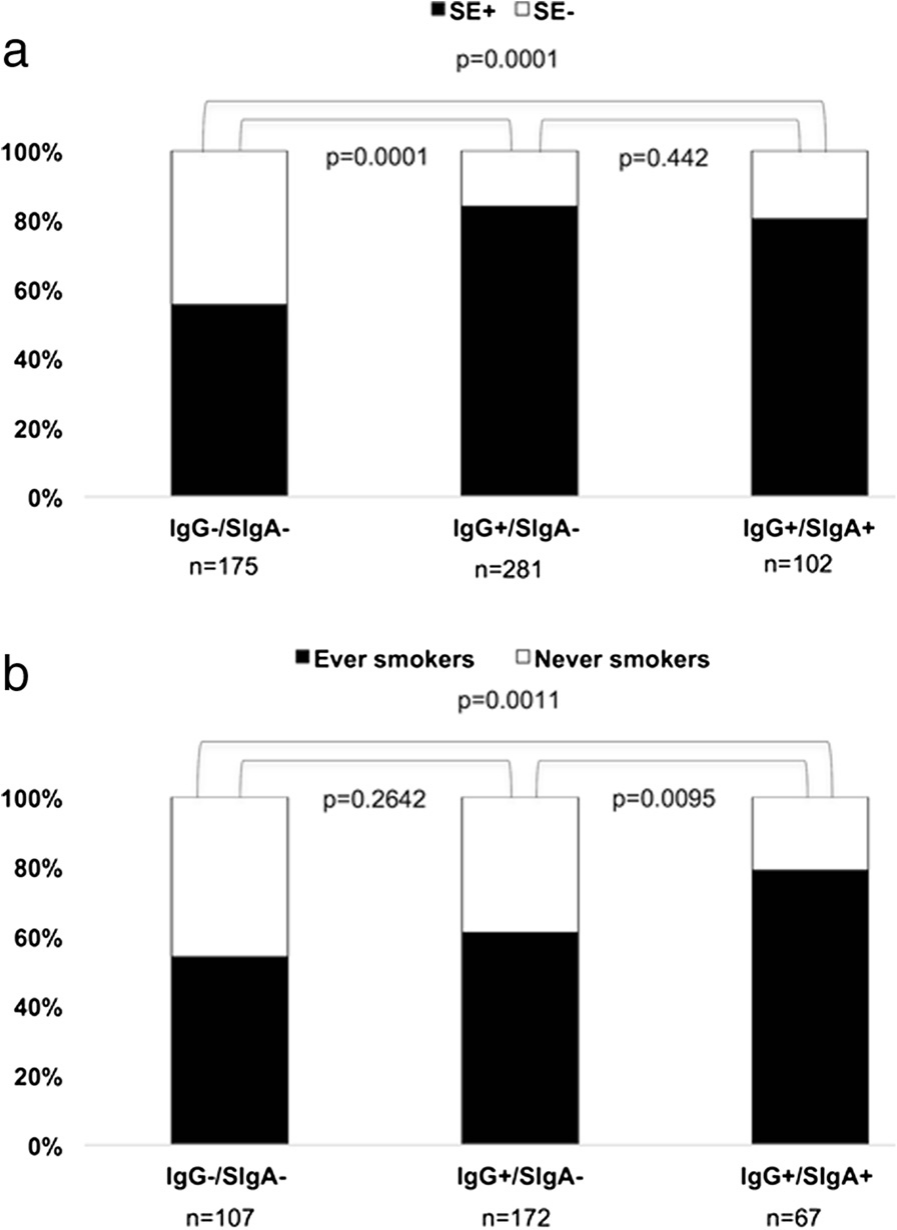
Secretory immunoglobulin A (SIgA) and immunoglobulin G (IgG) anticyclic citrullinated protein antibodies in relation to (**a**) shared epitope (SE) and (**b**) smoking in patients with early rheumatoid arthritis

At least one SE allele was present in 83 (80 %) of the SIgA anti-CCP patients and in 340 (73 %) of the SIgA anti-CCP-negative patients. As seen in Fig. 4a, carriage of SE was associated with IgG anti-CCP antibodies (*p* = 0.0001) but not with the co-occurrence of SIgA anti-CCP (*p* = 0.44). Smoking habits were analyzed in the same way as SE (Fig. 4b), where 53 (79 %) of the SIgA anti-CCP-positive patients were ever smokers, compared with 168 (59 %) of the SIgA-negative patients. Ever smoking was associated with the presence of SIgA anti-CCP antibodies (*p* = 0.0095), while IgG anti-CCP antibodies (in the absence of SIgA anti-CCP) was not (*p* = 0.26). Smoking was significantly associated with the presence of SIgA anti-CCP (OR 2.19, 95 % CI 1.01–4.37, *p* = 0.027), also after adjusting for age, sex, and SE (Table 2).

**Table 2:** Logistic regression analysis regarding secretory anti-cyclic citrullinated protein antibodies in patients with early rheumatoid arthritis

Variable	SIgA anti-CCP		Unadjusted		Adjusted	
	Positive	Negative	*p* value	OR (95 % CI)	*p* value	OR (95 % CI)
Ever smoking	49/63 (78 %)	94/156 (60 %)	0.015	2.31 (1.18–4.53)	0.027	2.19 (1.01–4.37)
One or two SE alleles	51/63 (81 %)	130/156 (83 %)	0.674	0.85 (0.40–1.81)	0.819	0.91 (0.42–2.00)
Female sex	44/63 (70 %)	117/156 (75 %)	0.434	1.30 (0.68–2.48)	0.814	1.09 (0.55–2.14)
Age at inclusion, years	58.7	52.6	0.002	1.04 (1.02–1.07)	0.005	1.04 (1.01–1.07)

*SE* Shared epitope, *SIgA* secretory immunoglobulin A, *CCP* cyclic citrullinated protein

Adjusted values are adjusted for all other variables in the table

## Discussion

In this study of well-characterized patients with early RA, we present the novel finding of circulating SIgA anti-CCP and describe its relationship to disease severity and etiological factors. Unlike IgG anti-CCP, circulating SIgA anti-CCP is not genetically related to SE, but is environmentally linked to cigarette smoking. This corresponds well to our previous studies regarding serum IgA ACPA, in which detection antibodies to α-chains were used and thus, as monomeric IgA predominates in the circulation, mainly concern nonsecretory IgA [16, 40]. Although secretory autoantibodies in sera from patients with other autoimmune diseases have been described earlier [32, 41], to the best of our knowledge this is the first report on SIgA ACPA in RA.

We found that approximately 25 % of patients with early RA with IgG class anti-CCP antibodies also had circulating SIgA anti-CCP antibodies, a proportion that is lower than IgA anti-CCP antibodies (39 %), which supports the notion that nonsecretory forms of IgA anti-CCP prevail in the circulation. The correlation between levels of SIgA anti-CCP and levels of IgG anti-CCP was found to be equally as strong as between SIgA and IgA anti-CCP. This somewhat surprising finding may suggest that mucosal immunization to citrullinated proteins is similarly important in systemic IgG and IgA ACPA responses in early RA. However, to enable firm conclusions regarding the temporal relationship between SIgA and other isotypes, serial analyses of preclinical cases are warranted. Also, it remains to be determined if and where mucosal immunization against citrullinated proteins occurs in IgG^+^/IgA^−^/SIgA^−^ patients.

On the basis of our previous findings of a milder disease in patients with RA with salivary IgA anti-CCP [13], we anticipated that circulating SIgA anti-CCP would associate with lower disease activity at the time of sampling, and possibly over time in early disease. Instead, it turned out that SIgA anti-CCP-positive patients had higher ESR and CRP levels at baseline than SIgA anti-CCP-negative patients, and the difference remained significant over 3 years regarding ESR. Although these findings are in line with previous reports on IgA RF, we did not find any significant predictive value of IgA anti-CCP in our cohorts. Intriguingly, while baseline inflammatory activity was higher in patients with SIgA anti-CCP antibodies, the number of swollen and tender joints was inversely related to SIgA anti-CCP antibody levels at the time of sampling. This finding opens up for speculation that the laboratory markers of inflammation partly reflect mucosal inflammation and not merely synovitis. Instead, the inverse correlation between antibody levels and effects on joints could hypothetically reflect the downregulating potential of antigen exposure in the gastrointestinal tract (i.e., oral tolerance). However, this remains speculative, as the origin of circulating SIgA anti-CCP remains to be determined. Further, it is possible that SIgA anti-CCP correlates with total IgA levels, which in turn may influence the ESR without being related to inflammation. In view of the small differences in disease activity measures over time as well as the lack of association with radiological progression, we see no clinical reason at present for routine baseline testing of SIgA anti-CCP. We recently reported that IgA and SIgA antiproteinase 3 (PR3) antibodies are more closely related to disease activity than IgG anti-PR3 in antineutrophil cytoplasmic antibody-associated vasculitis [42]. Analogously, future work should evaluate changes in SIgA anti-CCP levels in relation to therapeutic responses. Also, serial SIgA anti-CCP analyses in serum from pre-RA patients are of obvious interest regarding mucosal involvement in disease development.

Passive transport over leaky, inflamed mucosal membranes could be one possibility by which SIgA is translocated from mucosal secretions to the systemic circulation. However, there are several examples of active retransportation of SIgA across mucosal membranes from the luminal side to the subepithelial compartment [43, 44] and even inducing systemic immunity [45], but how SIgA is further transferred into the circulation remains unknown.

Total SIgA levels in the circulation are elevated in patients with RA compared not only with healthy individuals but also with patients with ankylosing spondylitis (i.e., another rheumatic disease with mucosal pathogenic implications) [32, 46]. In line with previous work regarding SIgA [46], we found no relationship between SIgA anti-CCP levels and use of NSAIDs, suggesting that iatrogenic damage to mucosal membranes is not a major cause of SIgA occurrence in the circulation. Cigarette smoking, on the other hand, appears robustly associated with serum SIgA anti-CCP antibody occurrence, even after considering the presence of IgG anti-CCP. Although not explicitly addressed in the present study, this finding may suggest that the airway mucosa is a major contributor to serum SIgA anti-CCP. Paired samples of serum and mucosal fluid from patients with RA will be crucial to delineating the origin of serum SIgA ACPAs and to determining the pathogenic role of each mucosal compartment.

## Conclusions

SIgA anti-CCP antibodies can be detected in serum from patients with RA and are associated with smoking and increased signs of systemic inflammation at the time of sampling.

## References

[1] Kroot EJ, de Jong BA, van Leeuwen MA, Swinkels H, van den Hoogen FH, van ’t Hof M (2000). The prognostic value of anti-cyclic citrullinated peptide antibody in patients with recent-onset rheumatoid arthritis. Arthritis Rheum..

[2] Snir O, Widhe M, Hermansson M, von Spee C, Lindberg J, Hensen S (2010). Antibodies to several citrullinated antigens are enriched in the joints of rheumatoid arthritis patients. Arthritis Rheum..

[3] Holers VM (2013). Autoimmunity to citrullinated proteins and the initiation of rheumatoid arthritis. Curr Opin Immunol..

[4] Nielen MM, van Schaardenburg D, Reesink HW, van de Stadt RJ, van der Horst-Bruinsma IE, de Koning MH (2004). Specific autoantibodies precede the symptoms of rheumatoid arthritis: a study of serial measurements in blood donors. Arthritis Rheum..

[5] Rantapää-Dahlqvist S, de Jong BA, Berglin E, Hallmans G, Wadell G, Stenlund H (2003). Antibodies against cyclic citrullinated peptide and IgA rheumatoid factor predict the development of rheumatoid arthritis. Arthritis Rheum..

[6] Kastbom A, Strandberg G, Lindroos A, Skogh T (2004). Anti-CCP antibody test predicts the disease course during 3 years in early rheumatoid arthritis (the Swedish TIRA project). Ann Rheum Dis..

[7] Rönnelid J, Wick MC, Lampa J, Lindblad S, Nordmark B, Klareskog L (2005). Longitudinal analysis of citrullinated protein/peptide antibodies (anti-CP) during 5 year follow up in early rheumatoid arthritis: anti-CP status predicts worse disease activity and greater radiological progression. Ann Rheum Dis..

[8] Kaur S, White S, Bartold PM (2013). Periodontal disease and rheumatoid arthritis: a systematic review. J Dent Res..

[9] Mikuls TR, Payne JB, Yu F, Thiele GM, Reynolds RJ, Cannon GW (2014). Periodontitis and *Porphyromonas gingivalis* in patients with rheumatoid arthritis. Arthritis Rheumatol..

[10] Hitchon CA, Chandad F, Ferucci ED, Willemze A, Ioan-Facsinay A, van der Woude D (2010). Antibodies to *Porphyromonas gingivalis* are associated with anticitrullinated protein antibodies in patients with rheumatoid arthritis and their relatives. J Rheumatol..

[11] Arvikar SL, Collier DS, Fisher MC, Unizony S, Cohen GL, McHugh G (2013). Clinical correlations with *Porphyromonas gingivalis* antibody responses in patients with early rheumatoid arthritis. Arthritis Res Ther..

[12] Harvey GP, Fitzsimmons TR, Dhamarpatni AASSK, Marchant C, Haynes DR, Bartold PM (2013). Expression of peptidylarginine deiminase-2 and -4, citrullinated proteins and anti-citrullinated protein antibodies in human gingiva. J Periodontal Res..

[13] Svärd A, Kastbom A, Sommarin Y, Skogh T (2013). Salivary IgA antibodies to cyclic citrullinated peptides (CCP) in rheumatoid arthritis. Immunobiology..

[14] Klareskog L, Stolt P, Lundberg K, Kallberg H, Bengtsson C, Grunewald J (2006). A new model for an etiology of rheumatoid arthritis: smoking may trigger HLA-DR (shared epitope)-restricted immune reactions to autoantigens modified by citrullination. Arthritis Rheum..

[15] Stolt P, Yahya A, Bengtsson C, Källberg H, Rönnelid J, Lundberg I (2010). Silica exposure among male current smokers is associated with a high risk of developing ACPA-positive rheumatoid arthritis. Ann Rheum Dis..

[16] Svärd A, Skogh T, Alfredsson L, Ilar A, Klareskog L, Bengtsson C (2015). Associations to smoking and shared epitope differ between IgA and IgG class antibodies to cyclic citrullinated peptides in early rheumatoid arthritis. Arthritis Rheumatol..

[17] Reynisdottir G, Karimi R, Joshua V, Olsen H, Hensvold AH, Harju A (2014). Structural changes and antibody enrichment in the lungs are early features of anti-citrullinated protein antibody-positive rheumatoid arthritis. Arthritis Rheumatol..

[18] Willis VC, Demoruelle MK, Derber LA, Chartier-Logan CJ, Parish MC, Pedraza IF (2013). Sputum autoantibodies in patients with established rheumatoid arthritis and subjects at risk of future clinically apparent disease. Arthritis Rheum..

[19] Ytterberg AJ, Joshua V, Reynisdottir G, Tarasova NK, Rutishauser D, Ossipova E (2015). Shared immunological targets in the lungs and joints of patients with rheumatoid arthritis: identification and validation. Ann Rheum Dis..

[20] Demoruelle MK, Weisman MH, Simonian PL, Lynch DA, Sachs PB, Pedraza IF (2012). Brief report: airways abnormalities and rheumatoid arthritis-related autoantibodies in subjects without arthritis: early injury or initiating site of autoimmunity?. Arthritis Rheum..

[21] Trentham DE (1998). Oral tolerization as a treatment of rheumatoid arthritis. Rheum Dis Clin North Am..

[22] Dorożyńska I, Majewska-Szczepanik M, Marcińska K, Szczepanik M (2014). Partial depletion of natural gut flora by antibiotic aggravates collagen induced arthritis (CIA) in mice. Pharmacol Rep..

[23] Ivanov WHJ, Darce J, Hattori K, Shima T, Umesaki Y (2010). Gut-residing segmented filamentous bacteria drive autoimmune arthritis via T helper 17 cells. Immunity..

[24] Vaahtovuo J, Munukka E, Korkeamäki M, Luukkainen R, Toivanen P (2008). Fecal microbiota in early rheumatoid arthritis. J Rheumatol..

[25] Dalvi S, Scher JU, Attur M, Patel J, Abramson SB (2012). Elevated fecal secretory immunoglobulin A, anti-cyclic citrullinated peptide antibodies, and cytokine levels in rheumatoid arthritis patients [abstract 1212]. Arthritis Rheum.

[26] Waldman RH, Mach JP, Stella MM, Rowe DS (1970). Secretory IgA in human serum. J Immunol..

[27] Brandtzaeg P (2013). Secretory IgA: designed for anti-microbial defense. Front Immunol..

[28] Eijgenraam JW, Oortwijn BD, Kamerling SWA, De Fijter JW, Van Den Wall Bake AWL, Daha MR (2008). Secretory immunoglobulin A (IgA) responses in IgA nephropathy patients after mucosal immunization, as part of a polymeric IgA response. Clin Exp Immunol..

[29] Berglin E, Johansson T, Sundin U, Jidell E, Wadell G, Hallmans G (2006). Radiological outcome in rheumatoid arthritis is predicted by presence of antibodies against cyclic citrullinated peptide before and at disease onset, and by IgA-RF at disease onset. Ann Rheum Dis..

[30] Lindqvist E, Eberhardt K, Bendtzen K, Heinegard D, Saxne T (2005). Prognostic laboratory markers of joint damage in rheumatoid arthritis. Ann Rheum Dis..

[31] Manfredsdottir VF, Vikingsdottir T, Jonsson T, Geirsson AJ, Kjartansson O, Heimisdottir M (2006). The effects of tobacco smoking and rheumatoid factor seropositivity on disease activity and joint damage in early rheumatoid arthritis. Rheumatology (Oxford).

[32] Jorgensen C, Moynier M, Bologna C, Youinou P, Sany J (1995). Rheumatoid factor associated with a secretory component in rheumatoid arthritis. Br J Rheumatol..

[33] Hallert E, Husberg M, Kalkan A, Rahmqvist M, Skogh T, Bernfort L (2015). Changes in sociodemographic characteristics at baseline in two Swedish cohorts of patients with early rheumatoid arthritis diagnosed 1996–98 and 2006–09. Scand J Rheumatol..

[34] Arnett FC, Edworthy SM, Bloch DA, McShane DJ, Fries JF, Cooper NS (1988). The American Rheumatism Association 1987 revised criteria for the classification of rheumatoid arthritis. Arthritis Rheum..

[35] Prevoo ML, van ’t Hof MA, Kuper HH, van Leeuwen MA, van de Putte LB, van Riel PL (1995). Modified disease activity scores that include twenty-eight-joint counts: development and validation in a prospective longitudinal study of patients with rheumatoid arthritis. Arthritis Rheum.

[36] Ekdahl C, Eberhardt K, Andersson SI, Svensson B (1988). Assessing disability in patients with rheumatoid arthritis: use of a Swedish version of the Stanford Health Assessment Questionnaire. Scand J Rheumatol..

[37] Olsson AR, Skogh T, Wingren G (2004). Aetiological factors of importance for the development of rheumatoid arthritis. Scand J Rheumatol..

[38] Larsen A (1995). How to apply Larsen score in evaluating radiographs of rheumatoid arthritis in long-term studies. J Rheumatol..

[39] Stolt P, Bengtsson C, Nordmark B, Lindblad S, Lundberg I, Klareskog L (2003). Quantification of the influence of cigarette smoking on rheumatoid arthritis: results from a population based case–control study, using incident cases. Ann Rheum Dis..

[40] Svärd A, Kastbom A, Reckner-Olsson A, Skogh T (2008). Presence and utility of IgA-class antibodies to cyclic citrullinated peptides in early rheumatoid arthritis: the Swedish TIRA project. Arthritis Res Ther..

[41] Iwasaki K, Okawa-Takatsuji M, Aotsuka S, Ono T (2003). Detection of anti-SS-A/Ro and anti-SS-B/La antibodies of IgA and IgG isotypes in saliva and sera of patients with Sjögren’s syndrome. Nihon Rinsho Meneki Gakkai Kaishi..

[42] Sandin C, Eriksson P, Segelmark M, Skogh T, Kastbom A (2016). IgA- and SIgA anti-PR3 antibodies in serum versus organ involvement and disease activity in PR3-ANCA-associated vasculitis. Clin Exp Immunol..

[43] Matysiak-Budnik T, Moura IC, Arcos-Fajardo M, Lebreton C, Ménard S, Candalh C (2008). Secretory IgA mediates retrotranscytosis of intact gliadin peptides via the transferrin receptor in celiac disease. J Exp Med..

[44] Rey J, Garin N, Spertini F, Corthesy B (2004). Targeting of secretory IgA to Peyer’s patch dendritic and T cells after transport by intestinal M cells. J Immunol..

[45] Favre L, Spertini F, Corthesy B (2005). Secretory IgA possesses intrinsic modulatory properties stimulating mucosal and systemic immune responses. J Immunol..

[46] Wendling D, Didier JM, Seilles E (1996). Serum secretory immunoglobulins in ankylosing spondylitis. Clin Rheumatol..

